# Two Extraordinary Forms of Rectal Fistula

**Published:** 1868-10

**Authors:** E. Andrews

**Affiliations:** Prof. of Principles and Practice of Surgery, Chicago Medical College


### CHICAGO MEDICAL EXAMINER.

N. S. DAVIS, M.D., EDITOR.

VOL. IX. OCTOBER, 1868. NO. 10.

### Original Contributions.

ARTICLE XXXVIII.

### TWO EXTRAORDINARY FORMS OF RECTAL FISTULA.

BY E. ANDREWS, M.D., Prof. of Principles and Practice of Surgery, Chicago Medical College.

I occasionally find practitioners of great general skill completely puzzled and confounded by the occurrence of unusual forms of intestinal fistula. The following examples are instructive in this connection:—

*Case* 1.—Annie B., æt. 12 years, was placed under my charge in the third stage of hip-disease. As she seemed likely to die of the irritation and suppuration, I exsected the carious head of the femur. This operation greatly improved her condition, so that she shortly began to recover flesh and strength.

At one of my visits, the mother stated to me that the child passed air from the intestines through the wound. Though rather incredulous on the subject, I tested the matter by injecting the rectum full of tepid water, when I found that it flowed out in a small but continuous stream from the incision by the trochanter. By making pressure at various places near the anus, I discovered a point where the fistulous canal crossed the tuberosity of the ischium, and where the touch of the finger would stop the flow of water from the wound. Taking a scalpel, I cut down upon this point, opened the fistula, and found that the water flowed freely out. Passing a probe along its track, towards the anus, I cut down again upon it, at a point pretty near the verge. Inserting the probe again at this point, I had no difficulty in following the sinus into the rectum, and effected a cure by cutting the sphincter in the usual manner.

*Case* 2.—Mrs. F. had an old femoral hernia, which at one time had terminated in an artificial anus in the groin, but had subsequently healed. I was some years afterwards called in for a new trouble. The whole circumference, almost, of the left hip was chronically swollen, and very tender. There was severe pain along the sciatic nerve, and great exhaustion and emaciation, which, unless relieved, was plainly destined to end in her death. On examination, the inflammation did not appear to be connected with the joint. The swelling fluctuated under the finger, and gave a distinct succussion upon coughing. Its entire circumference was resonant upon percussion, and pressure caused a gurgling noise, which proved the existence of air in the whole gluteal region, and also in the external part of the groin.

The case perplexed all who saw it, but was generally believed to be a hernia through the sciatic notch. On examination, I was dissatisfied with this explanation, because the tumor had no definite outline, like a true hernia, as well as because it was too tender, and quite irreducible. On inquiry, I found that the patient passed a considerable quantity of pus every day at stool, with some blood. Following up this hint, I injected the rectum full of air, and found that in this way I could easily fill up and distend the tumor, showing the connection between them.

With the concurrence of Dr. Ernz Schmidt, whose views coincided with mine, I determined to operate. The patient being placed under the influence of ether, I made an incision about half way between the trochanter and the border of the sacrum, down to the gluteus maximus muscle. Next carefully dividing the muscular tissue, as if coming down upon a hernial sac, I opened the cavity. There was no intestine present, but a large cavity, from which rushed a quantity of fetid gas, followed by some pus mingled with decayed fecal matter. Upon injecting the rectum full of tepid water, the fluid ran freely out of the wound. Bearing in mind the course of the fistula in the previous case, I carefully sought along the ischium and all adjacent parts for some point where pressure would stop the flow of water, but in vain; after a full exploration, it became evident that the sinus had a deeper course. Next passing a probe into the cavity, the fistula was found to run through the sciatic notch, above the greater ligament, and its opening, large enough to admit the point of the finger, could be felt on the side of the rectum by exploring that viscus. As there are too many important organs occupying the notch to allow of the enormous cut which would be required to open down to the anus, as in ordinary fistulas, it became necessary to devise other measures.

So far as I know, there is no published authority for a surgeon's guidance in such a case, and therefore it was requisite to act on general principles. I first enlarged the external incision towards the notch, so as to give as straight and short a fistula as possible, thus preventing the vast sac-like cavity from being any more distended, and giving it an opportunity to collapse and heal down as far as the edge of the incision. The next step was to introduce the index finger into the rectum, and grasping the sphincters between it and the thumb, to cut them through at one stroke. The retaining power of the anus being thus temporarily destroyed, I hoped that the contents of the rectum would pass straight downward, and give an opportunity for the fistula above to close.

The result surpassed my expectations. For a few days the sac continued to discharge its accumulation of old fæces, after which it sent forth clean pus, with occasionally a little flatus from the fistula. The sac collapsed, granulated rapidly, and was obliterated; the pain of the sciatic nerve ceased; the patient began to grow strong and fat, and in every way to present a striking improvement. At present, the fistula is almost entirely closed, but has occasionally bubbled out a little air.

It is contracting so rapidly that it seems likely to become completely obliterated without further interference. The patient is walking about, and even going out on visits, and is entirely freed from the danger which a few weeks ago threatened her life.

